# Digital Tools for People Without an Alzheimer Disease or Dementia Diagnosis: Scoping Review

**DOI:** 10.2196/64862

**Published:** 2025-08-25

**Authors:** Tanja J de Rijke, Thomas Engelsma, Chi Him Ng, Kyra K M Kaijser, Henk Herman Nap, Ellen M A Smets, Leonie N C Visser

**Affiliations:** 1 Department of Medical Psychology, Amsterdam University Medical Center, University of Amsterdam, Meibergdreef 9, Amsterdam, 1105 AZ, The Netherlands, 31 205663622; 2 Personalized Medicine, Amsterdam Public Health Research Institute, Amsterdam, The Netherlands; 3 Alzheimer Center Amsterdam, Department of Neurology, Amsterdam University Medical Center, Vrije Universiteit Amsterdam, Amsterdam, The Netherlands; 4 Neurodegeneration, Amsterdam Neuroscience Research Institute, Amsterdam, The Netherlands; 5 Digital Health, Amsterdam Public Health Research Institute, Amsterdam, The Netherlands; 6 eHealth Living & Learning Lab Amsterdam, Department of Medical Informatics, Amsterdam University Medical Center, University of Amsterdam, Amsterdam, The Netherlands; 7 Vilans Centre of Expertise for Long-Term Care, Utrecht, The Netherlands; 8 Human-Technology Interaction, Eindhoven University of Technology, Eindhoven, The Netherlands; 9 Quality of Care, Amsterdam Public Health Research Institute, Amsterdam, The Netherlands; 10 Division of Clinical Geriatrics, Center for Alzheimer Research, Department of Neurobiology, Care Sciences and Society, Karolinska Institutet, Stockholm, Sweden

**Keywords:** brain health, digital tools, eHealth, mHealth, stage of maturity, impact evaluation, future directions, implementation, equity, diversity, electronic health, mobile health

## Abstract

**Background:**

The field of Alzheimer disease (AD) has been moving toward earlier detection, personalized assessment of dementia risk, and dementia prevention. In the near future, a gap is expected between the growing demand for Alzheimer-related health care and a shrinking workforce. Responsibility is increasingly assigned to individuals to take an active role in their own brain health management and dementia prevention. Digital tools are thought to offer support regarding these processes.

**Objective:**

The aim of this scoping review is to create an overview of digital tools published in scientific literature in the context of AD and dementia aimed at people without an AD or dementia diagnosis as primary end users interacting with these digital tools. Additionally, we aim to gain insight into study sample diversity, the stage of maturity and evaluation of these tools, and recommended future directions.

**Methods:**

PubMed, IEEE Xplore, Ovid, and Web of Science were searched in January 2023, using terms related to AD and dementia, (pre-)disease stages, digital tools, and various purposes of digital tools. Two independent reviewers screened the titles and abstracts of 2811 records and subsequently 408 full-text articles, based on inclusion and exclusion criteria. Articles on tools targeting those with an AD or dementia diagnosis were excluded. Data extraction included information on the sample characteristics, the digital tool, stage of maturity and evaluation, and future (research) directions.

**Results:**

We included 39 articles, which were aimed at primary prevention (14/39, 36%), secondary prevention (11/39, 28%), daily life support (8/39, 21%), self-administered screening (4/39, 10%), or decision-making (2/39, 5%). Variation in the study sample emerged regarding cognitive abilities (healthy: 11/39, 28%; mild cognitive impairment: 12/39, 31%; [subjective] cognitive impairment: 9/39, 23%; “no dementia”: 1/39, 3%; and variation of cognitive abilities: 6/39, 15%). Less variation was found regarding sex (>50% female: 27/39, 69%), education (>50% high education: 13/39, 33%), and age (>50% >60 y: 23/39, 59%). Few articles reported on ethnicity (12/39, 31%) and digital literacy (11/39, 28%). Most tools were in an early evaluation and maturity stage (31/39, 80%), comprising preprototyping (1/35, 3%), prototyping (15/35, 43%), pilot testing (19/35, 54%), efficacy testing (18/40, 45%), usability testing (12/40, 30%), and feasibility testing (10/40, 25%). Future (research) directions comprised the need for further tool development, attention to diversity, and study advancements, such as large-scale longitudinal studies.

**Conclusions:**

Almost 80% of tools as reported on in academic literature are in early development comprising early stages of maturity and evaluation. Studies and evidence gathered for digital tools developed in the context of AD or dementia aimed at people without an AD or dementia diagnosis are thus preliminary and further development, research, and policy are required before these tools can be implemented for assessing, supporting, and preventing cognitive decline.

## Introduction

Dementia is a major public health problem [1]. Due to aging populations and the current lack of widely available effective therapeutic strategies, the number of people living with dementia is estimated to triple from 50 to 152 million by 2050 [2]. Alzheimer disease (AD) contributes greatly to these numbers, as AD is the most common cause of dementia [3]. Nowadays, biomarkers can detect AD years before clinical manifestation [4,5], implying that AD starts before symptom onset. Fourteen modifiable risk factors have been identified that account for around 45% of dementias worldwide that could be prevented or delayed [6,7]. This provides a window of opportunity to prevent or slow disease progression prior to the onset of dementia [8]. The field of AD and dementia research is therefore rapidly moving toward earlier detection of AD pathology, identification of (causal) risk factors, and better prediction of dementia to allow for timely, personalized dementia prevention among cognitively unimpaired people, people with subjective cognitive decline (SCD), and people with mild cognitive impairment (MCI) [9]. However, the aging population is associated with a growing prevalence of AD, and thus it is expected that there will be an increasing demand for AD-related health care, coinciding with a shrinking health care workforce [10]. The gap between health care demand and available workforce is expected to increase rapidly in the upcoming years. In addition, responsibility is increasingly assigned to individuals without an AD and dementia diagnosis to take an active role in their own brain health and dementia prevention [11-13].

Digital health tools can help mitigate the abovementioned growing gap and support people without an AD or dementia diagnosis to become more actively involved in the assessment and management of their brain health and care. Digital health tools are “smart devices and connected equipment that improve health,” such as mobile apps, digital platforms, or wearables [14]. These tools are considered to have relatively low development costs in comparison to analog tools or interventions, to be efficient, to be accessible for many, and to have the potential for high scalability [15,16]. Moreover, digital tools give people insight into their own health information, which may help people to enhance their understanding of their condition; give them control and responsibility; and help them make informed decisions [17]. Examples of digital tools that were developed for people visiting the memory clinic or cognitively unimpaired people in the context of AD diagnosis or dementia prevention include mobile or wearable technology to assess, detect, and monitor early-disease symptoms and predict progression to dementia; a digital question prompt list to support patient-clinician communication in the memory clinic; and a decision aid for people to decide whether or not to pursue a formal diagnostic workup for dementia [18-21].

However, the implementation and adoption of digital tools for people without an AD or dementia diagnosis is challenging [19,22,23]. Younger people may not perceive tools for brain health as relevant, because they are less concerned about the onset of dementia compared to older adults [24], who themselves may experience barriers that hamper digital tool use, such as limited digital literacy, declining cognitive skills, motor impairments, impaired vision or hearing, and comorbidities [3,25-27]. If people need to take a more active role in their own health and health care by using digital tools, usability and accessibility should be ensured. Many risk factors for dementia are related to existing inequities in accessing (digital) health, which occur more often in minority groups, such as people with a low socioeconomic position or people with low educational attainment [3,28], making ensuring the usability and accessibility of digital tools via an inclusive study sample during development particularly relevant for digital tools for those without an AD or dementia diagnosis. In addition, digital tools may not be fully developed to the extent that they are ready for large-scale implementation. The level of development of a digital tool can be determined on the basis of the stage of maturity and evaluation. The stage of maturity indicates how developed a digital tool is, whereas the stage of evaluation assesses implementation fidelity. Currently, insight is lacking regarding the stage of maturity and evaluation of digital tools aimed at people without an AD or dementia diagnosis. This limited understanding is hampering the assessment of the potential role of digital tools in the AD and dementia field. A synthesis of frequently mentioned future directions and current knowledge gaps could guide future research and development and the sustainable implementation of digital tools.

In this scoping review, we aim to review the current academic literature in the field of AD and dementia reporting on the design, development, evaluation, and implementation of digital tools for people without an AD or dementia diagnosis as primary end users interacting with the tool. In this paper, we intend to provide (1) a state-of-the-art overview of the available tools described in academic literature and their envisioned use, (2) insight into the included study samples and their diversity, (3) their level of development in terms of maturity and evaluation, and (4) a synthesis of future (research) directions as suggested in the reviewed articles.

## Methods

### Design and Registration

We conducted a scoping review following the PRISMA (Preferred Reporting Items for Systematic Reviews and Meta-Analyses) guidelines [29]. The final protocol was registered prospectively with the Open Science Framework on March 23, 2023. This study was conducted in the context of the ABOARD (A Personalized Medicine Approach for Alzheimer’s Disease) project, which aims for a future with personalized, patient-orchestrated diagnosis, prediction, and prevention of AD [30]. It is a large-scale research project carried out by a multidisciplinary consortium in the Netherlands.

### Eligibility Criteria and Selection Process

We defined digital health tools as “smart devices and connected equipment that improve health by having patients actively interact with them,” such as mobile apps, digital platforms, or wearables [14]. We searched online search engines and databases in January 2023, including PubMed, IEEE Xplore, Ovid, and Web of Science. The search strategy comprised terms related to AD and dementia, (pre-)disease stages, digital tools, and different purposes of digital tools (eg, “diagnosis,” “dementia prevention,” or “prediction”), and their synonyms). The query was co-developed with the research team. The final search queries for the various engines and databases can be found in Multimedia Appendix 1. Definitive search results (ie, records) were exported to Rayyan software [31]. Duplicate records were automatically identified by Rayyan, and potential missing duplicates were manually identified by the researchers and subsequently removed. Two researchers (CHN, TJdR) independently reviewed all identified records based on our inclusion and exclusion criteria. When in doubt, an extra researcher (TE) reviewed these records as well. Articles were included if they described research on a digital tool developed in the field of AD or dementia, with people without an AD or dementia diagnosis as the intended primary end users, that is, those interacting with the tool. Additional inclusion criteria comprised full text availability, peer-reviewed articles written in English or Dutch, the articles addressing humans (not animals), and articles containing original empirical research data. As the aim of this study is to provide a state-of-the-art overview of recent digital tools reported on in academic literature, we applied a 10-year filter (2013‐2023) to include relevant tools while excluding outdated technological developments (Textbox 1).

Textbox 1. **Inclusion criteria.**

- Research on a digital tool developed in the context of Alzheimer disease or dementia
- Primary end users comprise people without an Alzheimer disease or dementia diagnosis, that is, cognitively unimpaired people and/or people with subjective cognitive decline or mild cognitive impairment
- Articles published in 2013-2023 (10-year filter)
- Peer-reviewed articles written in English or Dutch
- Articles containing original empirical research data
- Article addresses humans (not animals)
- Full text availability

Titles and abstracts were reviewed on these inclusion criteria. For the remaining set, full-text articles were screened. The researchers compared their screening decisions and discussed these until they reached consensus.

### Data Extraction

A data extraction sheet was developed based on our aims. For the overview of available tools, we (CHN, KKMK, TJdR) extracted information on the name of the tool, purpose of the tool, place of use (ie, home or clinical setting), and self-guidance (ie, fully self-guided or researcher/clinician-assisted). Data on study samples (ie, the total number of participants, cognitive status, age, sex, educational attainment, ethnicity, and digital literacy) were extracted by KKMK and checked by TJdR. We extracted data that were explicitly mentioned in the demographic section of an article, as well as data that were implicitly mentioned in the inclusion and exclusion criteria of the article (eg, if digital literacy was an inclusion criterion, we assumed that the sample was probably digitally literate). Data regarding the stage of developmental maturity and stage of evaluation were extracted and categorized (TJdR) in accordance with the World Health Organization guide [32]; in case of doubt, these were discussed with others (TE, LNCV, EMAS). Early maturity stages comprise a preprototype, prototype, or pilot product [32]. Mid-maturity comprises the demonstration phase that assesses the costs and implementation requirements, in which the effect of the tool is tested in an uncontrolled situation limited to a certain population or geography [32]. The advanced stage of maturity comprises scale-ups (ie, tools that are ready to be optimized and scaled up across multiple [sub]national and population levels) or integrated and sustained programs (ie, integrating tools in a broader health system and determining components of the enabling environment to maximize impact at a large scale) [32]. Stage of evaluation is related to the aforementioned stages of maturity and informs on the level of evidence for a digital tool, thus informing on implementation fidelity. The early stage of evaluation comprises feasibility (ie, assessing if the tool works in a given context), usability (ie, assessing if the tool can be used by users), and efficacy testing (ie, assessing if the tool can achieve the intended results in a controlled setting) [32]. Mid-stage evaluation encompasses effectiveness testing, which informs if the tool can achieve the results in a noncontrolled setting [32]. The late stage of evaluation addresses implementation issues regarding the tool’s uptake, integration, and sustainability in a given context, including policies and practices [32]. If multiple stages of maturity and evaluation were present, we chose the most advanced stage (eg, if early and mid-maturity were described, we categorized the study as mid-mature). In this study, we assessed the stage of maturity and stage of evaluation of a tool in relation to a specific study context and population as described in a scientific article. For instance, a Wii is a mature digital tool by itself, but it was evaluated as an early-stage tool when the scientific article was a pilot study among people with cognitive complaints that assessed the usability of the tool. We (KKMK, TJdR) also extracted data on future directions bottom-up from Future Research or Future Directions sections in the Discussion section of the included articles.

### Data Synthesis

Next, a narrative synthesis on the main findings in relation to our main objectives was written by TJdR. We grouped the included articles by tool category (ie, primary prevention, secondary prevention, daily life support, self-administered screening, and decision support). We defined dementia prevention tools as tools that aim for primary (healthy people and people with SCD) and secondary dementia prevention (people with MCI), including both single- and multidomain interventions. Daily life support tools comprised tools that aim to support daily life for people living with cognitive complaints, enhance overall well-being in relation to the cognitive complaints, and improve quality of life. The category self-administered screening tools comprised self-administered AD/dementia-risk assessment tools and tools that assess cognitive functioning, including memory, language, and perception. These types of tools may involve questions and/or tasks to complete that could indicate whether there is any ground for concern and do not replace a detailed clinical assessment required for a formal diagnosis of dementia or MCI [33]. Decision support tools consist of tools containing a health care–related decision aid for people without an AD or dementia diagnosis. Subthemes resulting from the data extraction of reported future directions were grouped into overarching themes.

## Results

### Search Findings

**Figure 1.**
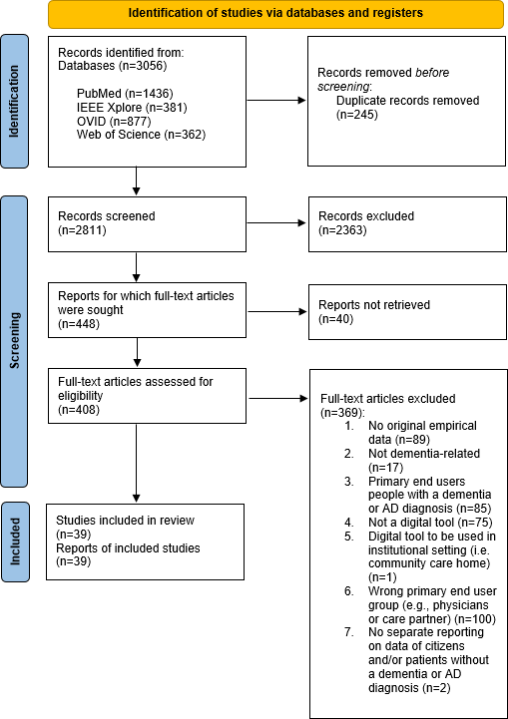
PRISMA 2020 flow chart of included articles. PRISMA: Preferred Reporting Items for Systematic Reviews and Meta-Analyses.

The search yielded a total of 3056 records, of which 2811 records remained after removing duplicates. After screening the titles and abstracts, 408 articles were subjected to full-text screening. Finally, 39 articles met the inclusion criteria and were found suitable for data extraction (Figure 1).

### Study Samples

The following study populations could be identified: people with MCI (12/39, 31%), cognitively healthy people (11/39, 28%), (subjective) cognitive impairment (9/39, 23%), or “no dementia” (1/39, 3%). Six articles (15%) included people with a variety of cognitive abilities (Table 1). Most articles included people aged 60 years and older (>50% >60 years: 23/39, 59%), with more female representation (>50% female: 27/39, 69%) and highly educated study participants (>50% high education: 13/39, 33%; Table 1). Twelve articles (12/39, 31%) reported data on the ethnic background of participants. These articles predominantly included White people or those of European ancestry (Table 1). Of the 39 articles, 11 (28%) explicitly discussed the digital literacy levels of their study participants. These articles revealed that the majority of their samples consisted of individuals proficient in digital skills. In 2 articles, around half of the participants lacked experience in using digital technologies (Table 1). Regarding the inclusion and exclusion criteria of the included articles, we found some articles (9/39, 23%) mentioning digital literacy and/or having internet access and/or the possession of a smartphone, tablet, or computer as an inclusion criterion [34-42]. None of the articles mentioned educational attainment or ethnicity as an inclusion criterion. For one study, the recruitment was done fully online, potentially resulting in a sample of people with sufficient digital literacy skills [43].

**Table 1.** Study participant characteristics of the included articles per tool category.

	Total, n	Cognitive status	Age (mean, SD) or age group (%)	Sex, % female	Ethnicity (%)	Educational level (%, n, or mean, SD)	Digital literacy
**Primary prevention**							
Aalbers et al (2016); the Netherlands [37]	Goal-setting group (n=1212) Nongoal setting group (n=1093)	Healthy	Goal-setting group: 52.3 (12.2) Nongoal setting group: 51.3 (13.7)	Goal-setting group: 71.1% Nongoal setting group: 63%	N/A^a^	Goal setting: secondary or lower (32.3%); vocational degree (44.3%); university degree (23.4%) No goal setting: secondary or lower (37.3%); vocational degree (39.5%); university degree (23.3%)	N/A
Anstey et al (2020); Australia [40]	BBL-GP^b^ (n=42) LMP^c^ (n=41) AC^d^ (n=42)	Healthy	BBL-GP: 51.1 (14.2) LMP: 51.4 (11.7) AC: 49.9 (14)	BBL-GP: 67% LMP: 66% AC: 74%	N/A	Years of education: BBL-GP: 15.5 (4.5) LMP: 16.4 (4.3) AC: 16.1 (4.2)	N/A
Bird et al (2021); United Kingdom [44]	Participants (n=4826)	Healthy	Age 50‐55 (33.7%) Age 56‐60 (32.3%) Age 61‐65 (28.8%)	73%	White (86.7%) Asian (1.1%) African (0.4%) Other (6%)	Undergraduate (26.2%) Postgraduate (22.7%) Diploma (16.4%) A-level (10.3%) General Certificate of Secondary Education (12.5%) Trade certificate (5.8%) Other (1.2%)	N/A
Bott et al (2018); United States [39]	Participants (n=82)	SCD^e^	64 (4)	24%	African American (6%) Asian (1%) White (88%) Hispanic (4%) Other (1%)	High school (4%) Vocational training (2%) Some college, no degree (27%) Bachelor’s degree (35%) Graduate degree (23%) Doctorate (9%)	Inclusion criteria: Ability to make and receive phone calls Send and receive text messages Access desktop computer and video teleconferencing
Corbett et al (2015); United States [36]	ReaCT^f^ (n=2557) GCT^g^ (n=2432) Control (n=1753)	Healthy	ReaCT: 58.5 (6.5) GCT: 59.1 (6.4) Control: 59.1 (6.6)	ReaCT: 68% GCT: 68% Control: 62%	ReaCT: White (96.9%) GCT: White (97%) Control: White (97.4%)	ReaCT university: 51.7% GCT university: 50.6% Control university: 47.4%	Experienced as all contact was via email/online
Eun et al (2022); South Korea [45]	Participants (n=37)	Healthy	Under 70 (17.9%) 70‐74 (35.9%) 75‐79 (30.8%) Over 80 (15.4%)	87.2%	N/A	N/A	N/A
Glenn et al (2019); Japan [38]	Healthy adults (n=242)	Healthy	Females: 51 (7.9) Males: 51 (1)	51.7%	N/A (Japanese language)	N/A	N/A
Isaacson et al (2014); United States [43]	Participants (n=100)	Healthy	30s (5.5%) 40s (12.2%) 50s (43.3%) 60s (21.1%) 70s (16.6%) 80s (3.3%) 90s (1.1%)	79.8%	N/A	High school/secondary (40.2%) Postgraduate (24.1%) Associate degree (18.4%) Bachelor (16.1%)	N/A
Oh et al (2018); South-Korea [46]	SMART^h^ (n=18) AC (n=19) Wait-list (n=16)	SCD	SMART: 59.3 (5.1) AC: 58.8 (5) Wait-list: 58.8 (5)	SMART: 50% AC: 53% Wait-list: 56%	Korean (100%)	Education in years: SMART: 14.2 (3.7) AC: 14.2 (2.3) Wait-list: 13.4 (3.6)	N/A
Palac et al (2019); United States [47]	Intervention (n=14) Control (n=13)	SCD	Intervention: 49.4 (5.1) Control: 52.2 (4.7)	Intervention: 85.7% Control: 69.2%	Intervention: White (64.3%); African American (21.4%); Asian (7.1%) Control: White (69.2%); African American (15.4%); Asian (7.7%); American Indian (7.7%)	College degree: Intervention (85.7%) Control (76.9%)	N/A
Petrella et al (2023); United States [48]	Intervention (n=39) Control (n=48)	SCD	Intervention: 69.3 (8.1) Control: 70.8 (8.7)	Total: 58% Intervention: 71.8% Control: 45.8%	African American (30%)	Years of education: Intervention: 16.74 (3.03) Control: 17.06 (3.19)	N/A
Tedim Cruz et al (2014); Portugal [49]	Participants (n=45)	SCD	50.7 (17)	35.6%	N/A	Years of education: 7.8 (4.9)	N/A
Vanoh et al (2018); Malaysia [34]	Healthy older people (n=30)	Healthy	65.1 (3.8)	60%	Malay (30%) Indian (30%) Chinese (30%)	College/university (76.7%) Secondary education (20%) Incomplete secondary education (3.3%)	Yes (76.7%), no (20%)
Wesselman et al (2020); the Netherlands and Germany [41]	SCD (n=137)	SCD	65.1 (8.6)	57%	N/A	Years of education: 11.3 (1.9)	All have a smartphone, tablet, or computer
**Secondary prevention**							
Bahar-Fuchs et al (2017); Australia [50]	MCI^i^ (n=9) MrNPS^j^ (n=11) 25 MCI+ (21 intervention CCT^k^ vs 23 active control)	MCI MrNPS	CCT: 74 (8) AC: 75.3 (5.8) MCI: 74.7 (6.8) MrNPS: 71.5 (7.4) MCI+: 76.0 (6.3)	63.3%	N/A	CCT: 14.4 (3.2) AC: 14.5 (3) MCI: 14.8 (2.6) MrNPS: 13.7 (2.6) MCI+: 14.7 (3.4)	N/A
Djabelkhir et al (2017); France [51]	MCI in CCE^l^ group (n=10) MCI in CCS^m^ group (n=10)	MCI	CCE: 78.2 (7) CCS: 75.2 (6.4)	CCE: 60% CCS:70%	N/A	CCE college or higher: 44.4% CCS college or higher: 60%	N/A
Hartin et al (2016); United States [35]	Intervention (n=102) Control (n=42)	No dementia	N/A	N/A	N/A	N/A	N/A
Hassandra et al (2021); Greece [52]	Study 1: MCI (n=16) SCD (n=4) Study 2: Undergraduates (n=30) MCI (n=27)	Healthy SCD MCI	Study 1 MCI/SCD: 76.3 (5) Study 2: Undergraduates: 20.9 (1.2) MCI: 73.2 (9.3)	Study 1: MCI/SCD (75%) Study 2: Undergraduates (53.3%) MCI (70.1%)	N/A	Study 1: MCI/SCD mean years of education 11.35 (5.76) Study 2: Undergraduates (high level of education: 100%) MCI (primary: 31%; secondary: 42%; higher: 27%)	Study 1: MCI/SCD (phone user never: 4%; computer use never: 44%; gaming use never: 55%) Study 2: Undergraduates (phone user never: 0%; computer use never: 20%; gaming use never: 73%)
Hill et al (2018); United States [53]	Participants (n=12)	MCI	79 (4.2)	58%	White (92%)	Years of education: High school (8%) Some college, no degree (25%) Bachelor or higher (50%)	N/A
Hughes et al (2014); United States [54]	Intervention (n=10) Control (n=10)	MCI	Intervention: 78.5 (7.1) Control: 76.2 (4.3)	Intervention: 80% Control: 60%	Intervention: White (70%) Control: White (90%)	Intervention, years of education: 13.8 (2.4) Control, years of education: 13.1 (1.9)	All had experience with Nintendo Wii
Infarinato et al (2020); Italy [55]	MCI (n=15)	MCI	71.9 (0.9)	53%	N/A	N/A	N/A
Lin et al (2022); Taiwan [56]	Intervention (n=8) Control (n=8)	MCI	Intervention: 79.8 (4.9) Control: 77.8 (6.7)	Intervention: 62.5% Control: 62.5%	N/A	Intervention: >junior high (50%) Control: >junior high (25%)	N/A
Savulich et al (2017); United Kingdom [57]	Intervention group aMCI^n^ (n=21) Control group aMCI (n=21)	MCI	Intervention group: 75.2 (7.4) Control group: 76.9 (8.3)	Intervention group: 47% Control: 33%	N/A	Intervention, age left education: 15.9 (1.3) Control, age left education: 16.0 (2.1)	Internet use (h/wk): Intervention: 2.2 (6.6) Control: 2.3 (4.5)
Smith et al (2020); United States [58]	Healthy older adults (n=5) Older adults with MCI (n=5)	Healthy MCI	Total: 74 Healthy older adult: 73 MCI: 75	30%	N/A	N/A	N/A
Zajaç-Lamparska et al (2019), Poland [59]	Older adults without dementia (n=72) Older adults with mild dementia (n=27)	Healthy older adults Mild dementia (Mini Mental State Examination: 22.33, 1.21)	Older adults without dementia: 67.9 (5.8) Older adults with mild dementia: 72 (7.4)	Older adults without dementia (75%) Older adults with mild dementia (81.5%)	N/A	Years of education: Older adults without dementia: 13.6 (3.9) Older adults with mild dementia: 12.6 (3.3)	N/A
**Daily life support**							
Baric et al (2019); Sweden [42]	Participants (n=20)	Healthy	73.7 (5.2)	45%	N/A	Years of education: 6‐9 (35%) 12‐14 (35%) University (40%)	Daily/weekly computer use (55%) Daily/weekly mobile phone use (85%)
Beentjes et al (2023); the Netherlands [60]	Experimental group (n=28) Control (n=31)	MCI	Experimental: 72.7 (7.8) Control: 71.7 (9.6)	Experimental (43%) Control (35%)	N/A	Experimental: primary (0%), secondary vocational (28%), secondary academic (4%), further education vocational (25%), high education vocational (29%), high education academic (14%) Control: primary (3%), secondary vocational (19%), secondary academic (3%), further education vocational (29%), high education vocational (39%), high education academic (6%)	Never: Experimental (46%) Control (52%)
Chudoba et al (2020); United States [61]	Case reports (n=3)	SCD	Case 1: 69 Case 2: 39 Case 3: 72	Case 1 (female) Case 2 (female) Case 3 (female)	N/A	Case 1 (14 years of education) Case 2 (13 years of education) Case 3 (13 years of education)	N/A
Cortellessa et al (2021); Spain and Romania [62]	Participants (n=90) with informal carers and health care professionals	Cognitive impairment (23‐27 Mini-Mental State Examination) or self-perceived cognitive impairment or caregivers’ perception of cognitive impairment that has been present for more than 6 months	N/A	N/A	N/A	N/A	N/A
Piculell et al (2021); Sweden [63]	Participants (n=16)	Cognitive impairment (Mini-Mental State Examination score ranging from 20‐26)	71‐100 years	25%	N/A	Elementary (n=6) Secondary (n=2) Senior high school (n=5) University (n=2)	N/A
Schmitter-Edgecombe et al (2022); United States [64]	Adults who met criteria for amnestic MCI (n=32)	MCI	Partnered: 74.4 (5.6) EMMA^o^: only 70.6 (6.3)	71% partnered 33% EMMA only	N/A	Partnered: 17.1 (2.2) EMMA only: 15.6 (2)	Technology comfort: Partnered: 3.6 (1.0) EMMA only: 4.7 (1.2)
Scullin et al (2022); United States [65]	Reminder app (n=23) Digital recorder app (n=25)	MCI	Reminder app: 73.2 (6) Digital recorder app: 76.4 (8)	Reminder app (52%) Digital recorder app (32%)	Non-Caucasian: Reminder app (22%) Digital recorder app (12%)	Years of education: Reminder app: 14.5 (2.5) Digital recorder app: 14.8 (2.4)	N/A
Quintana et al (2020); Sweden and Spain [66]	Sweden: MCI (n=9) Carers (n=9) Spain: MCI (n=10) Carers (n=10)	MCI	Sweden: MCI: 77 Carers: 68 Spain: MCI: 80 Carers: 64	Sweden: MCI (33%) Carers (45%) Spain: MCI (50%) Carers (30%)	N/A	N/A	Smartphone/tablet use every day: Sweden: people with MCI (55%) Spain: people with MCI (70%)
**Self-administered screening**							
Bonnechère et al (2018); Belgium [67]	Young (n=20) Healthy older adults (n=27) Cognitive impairment (n=29)	Cognitive impairment (Mini-Mental State Examination=20‐24) Healthy	Young: 26 (3) Healthy older adults: 47 (10) Cognitive impairment: 80 (12)	N/A	N/A	N/A	N/A
Brandt et al (2014); United States [68]	Study 1: older adults (n=116) Study 2: adult dementia (n=50); nondementia (n=44)	Healthy MCI AD^p^ Mood disorder Other psychiatric disease Other type of dementia	Study 1: Older adults: 78.4 (8.4) Control: 73.2 (8.5) Study 2: Dementia: 78.24 (8.7) Nondementia: 74.8 (7.9)	Study 1: Older adults: 78% Control: 78% Study 2: Dementia: 74% Nondementia: 59%	N/A	Study 1: Older adults: 14.1 (2.3) Controls: 14.3 (2.7) Study 2: Dementia 12.9 (2.9) Nondementia: 14.5 (2.9)	N/A
Brandt et al (2013); United States [69]	Anonymous internet sample (n=4125) Clinical sample (n=52)	Healthy MCI Probable AD Non-AD dementia	Anonymous internet sample: 57.2, 13.2 Clinical sample: 75.9, 3	Internet sample: 68.1% Clinical sample: 64%	N/A	Internet sample, mean highest grade completed: 15.7, 2.7 Clinical sample, mean highest grade completed: 13.8, 3	N/A
Lancaster et al (2020); United Kingdom [70]	Participants (n=35)	Healthy	52.6 (5.1)	74.0%	N/A	15.5 (2.7)	N/A
**Decision support**							
Bogza et al (2020); Canada [71]	HCPs^q^ (n=7) People with MCI (n=12)	MCI	HCPs: <age 30 (14%), age 30‐39 (57%), age 50‐59 (29%) MCI: age 60‐64 (17%), age 65-74 (50%), age 75-84 (25%), age >85 (8%)	HCP (86%) MCI (50%)	HCP: N/A MCI: White (100%)	HCP: N/A MCI: no education (8%), high school degree (33%), college degree (25%), university degree (25%)	N/A
Ekstract et al (2017); United States [72]	Participants (n=1262)	Healthy	54.5 (range: 22-87)	86%	White (90%)	N/A	N/A

a N/A: not applicable.

b BBL-GP: Body Brain Life in General Practice.

c LMP: Lifestyle Modification Program.

d AC: active control.

e SCD: subjective cognitive decline.

f ReaCT: reasoning cognitive training.

g GCT: general cognitive training.

h SMART: Specific, Measurable, Attainable, Realistic, and Time-bound Goal Enhanced Debriefing Group.

i MCI: mild cognitive impairment.

j MrNPS: mood-related neuropsychiatric symptoms.

k CCT: computerized cognitive training.

l CCE: computerized cognitive engagement.

m CCS: computerized cognitive stimulation.

n aMCI: amnestic mild cognitive impairment.

o EMMA: electronic memory and management aid.

p AD: Alzheimer disease.

q HCP: health care professional.

### Study Characteristics

Table 2 provides an overview of the included articles, together with a description of the author, year of publication, country, name of tool, purpose of tool, type of tool, fully self-guided (yes/no), home setting (yes/no), stage of maturity, and stage of evaluation. Almost 70% of these tools have been developed since 2018 (27/39, 69%). A variety of types of tools was found, including web-, tablet-, or smartphone-based apps (n=25); websites (n=12); and game consoles or video games (n=2) (Table 2). Tools were mainly used in the home setting (n=30) and fully self-guided (n=30) (Table 2). If tools were used in a clinical setting (n=9; Table 2), this was usually under the supervision of a health care professional or researcher.

**Table 2.** Types of digital tools per tool category.

Study	Name and purpose of the tool	Type of tool	Fully self-guided	Home setting	Stage of tool maturity in the context of the article [32]	Stage of tool evaluation in the context of the article [32]
**Primary prevention**						
Aalbers et al (2016); the Netherlands [37]	BAM-COG: Motivate adults to adopt healthy lifestyle changes to prevent cognitive decline	Internet-based game test battery (website)	Yes	Yes	Mid (demonstration)	Effectiveness
Anstey et al (2020); Australia [40]	BBL-GP: Reduce risk of cognitive decline in at-risk individuals	Internet-based online environment (website)	Yes	Yes	Early (pilot)	Efficacy
Bird et al (2021); United Kingdom [44]	eCFT: Behavior changes; promote cognitive-healthy lifestyle	Website	No	No	Early (prototype)	Usability
Bott et al (2018); United States [39]	VC Health: Improve cognitive abilities; reduce depression/anxiety; prompt lifestyle behavior changes	Internet-based online environment (website)	Yes	Yes	Mid	Effectiveness
Corbett et al (2015); United States [36]	ReaCT and GCT: Improve cognitive abilities, dementia prevention, and maintenance of cognitive function	Computerized cognitive training (website)	Yes	Yes	Early (pilot)	Efficacy
Eun et al (2022); South Korea [45]	Artificial intelligence–based serious game: Enhance participants’ engagement in cognitive training	Web-based app; artificial intelligence–based; mobile app	Yes	Yes	Early (prototype and pilot)	Efficacy
Glenn et al (2019); Japan [38]	Neurotrack MHP: Change behavior to improve risk factors related to cognitive decline	Smartphone app	Yes	Yes	Early (pilot)	Feasibility
Isaacson et al (2014); United States [43]	Alzheimer’s Universe: Impact knowledge and behavior change	Website	Yes	Yes	Mid (demonstration)	Effectiveness
Oh et al (2018); South Korea [46]	SMART: Improve attention and memory performance in older adults with subjective memory complaints	Smartphone app	Yes	Yes	Mid (demonstration)	Effectiveness
Palac et al (2019); United States [47]	BitGym: Improve physical activity, increase wayfinding self-efficacy and performance	iPad app on aerobic machine	Yes	No	Early (pilot)	Feasibility and efficacy
Petrella et al (2023); United States [48]	Lumosity: Improve cognitive abilities; stimulate cognitive domains	Web-based app	Yes	Yes	Early (pilot)	Efficacy
Tedim Cruz et al (2014); Portugal [49]	COGWEB: Enhance cognitive functioning	Computerized cognitive training (website)	Yes	Yes	Mid (demonstration)	Effectiveness
Vanoh et al (2018); Malaysia [34]	WESIHAT 2.0: Educate older adults about precautionary strategies against MCI^a^	Web-based app	Yes	Yes	Early (prototype)	Usability
Wesselman et al (2020); the Netherlands and Germany [41]	Hello Brain: Enhance brain-healthy lifestyle	Web-based app (smartphone, tablet, computer)	Yes	Yes	Early (prototype)	Feasibility and usability
**Secondary prevention**						
Bahar-Fuchs et al (2017); Australia [50]	CogniFit General Training: Improve cognitive abilities	Computerized cognitive training (website)	Yes	Yes	Early (pilot)	Efficacy
Djabelkhir et al (2017); France [51]	CCS and CCE: Improve cognitive abilities; stimulate cognitive domains	Tablet app and television	No	No	Early (preprototype and prototype)	Feasibility
Hartin et al (2016); United States [35]	Gray Matters: Promote and monitor behavior change and encourage the motivations of the participants	Smartphone app; tablet app	Yes	Yes	Early (prototype and pilot)	Feasibility, usability, and efficacy
Hassandra et al (2021); Greece [52]	VRADA: Improve cognition and physical fitness of people with MCI	Virtual reality–based app	No	No	Early (prototype)	Usability
Hill et al (2018); United States [53]	Modified ATA: Enhance cognitive functioning	Tablet app	Yes	Yes	Early (prototype)	Usability
Hughes et al (2014); United States [54]	Nintendo Wii: Improve cognitive performance	Nintendo Wii	No	No	Early (pilot)	Feasibility and efficacy
Infarinato et al (2020); Italy [55]	EWall: Improve physical and mental health	Television/touchscreen-based app	Yes	Yes	Early (prototype)	Usability
Lin et al (2022); Taiwan [56]	Xavix Hot Plus: Improve cognitive abilities	Video games	No	No	Early (pilot)	Feasibility and efficacy
Savulich et al (2017); United Kingdom [57]	Game Show: Improve cognitive abilities	Tablet app	Yes	No	Early (pilot)	Efficacy
Smith et al (2020); United States [58]	mPACT: Stimulate cognitive abilities via physical activity	Tablet app	Yes	Yes	Early (prototype)	Usability
Zajaç-Lamparska et al (2019); Poland [59]	GRADYS: Cognitive intervention or stimulation	Virtual reality–based	No	No	Early (pilot)	Feasibility and efficacy
**Daily life support**						
Baric et al (2019); Sweden [42]	RemindMe: Aid people with active reminders	Smartphone app; SMS text messaging	Yes	Yes	Early (pilot)	Usability
Beentjes et al (2023); the Netherlands [60]	FindMyApps: Aid people with mild dementia/MCI and caregivers to find user-friendly apps	Web app for tablet or smartphone	No	Yes	Early (pilot)	Feasibility and efficacy
Chudoba et al (2020); United States [61]	RBANS (DMN): Help maintain functional independence and quality of life via digital memory notebook	App	Yes	Yes	Mid (demonstration)	Effectiveness
Cortellessa et al (2021); Spain and Romania [62]	TV-AssistDem: Facilitate remote support and communication between patients, caregivers, and health care professionals	Television-based app	Yes	Yes	Early (prototype)	Feasibility
Piculell et al (2021); Sweden [63]	SMART4MD: Facilitate sense of coherence	Tablet app	Yes	Yes	Early (prototype)	Usability
Schmitter-Edgecombe et al (2022); United States [64]	EMMA: Mitigate impact of cognitive impairment on daily activities	App	No	Yes	Mid (demonstration)	Effectiveness
Scullin et al (2022); United States [65]	Reminder App: Support prospective memory in people with MCI and mild dementia	Smartphone-based app	Yes	Yes	Early (pilot)	Feasibility and efficacy
Quintana et al (2020); Sweden and Spain [66]	SMART4MD: Support people with cognitive impairment and carers to improve quality of life	Tablet app or smartphone app	Yes	Yes	Early (prototype)	Feasibility and usability
**Self-reported cognitive and risk assessment**						
Bonnechère et al (2018); Belgium [67]	MG: Assess cognitive abilities	Tablet app/touch pad	Yes	Yes	Early (pilot)	Efficacy
Brandt et al (2014); United States [68]	Dementia risk assessment: Assess risk of dementia	Web-based website	Yes	Yes	Early (pilot)	Efficacy
Brandt et al (2013); United States [69]	Dementia risk assessment: Assess risk of dementia	Web-based website	Yes	Yes	Early (pilot)	Efficacy
Lancaster et al (2020); United Kingdom [70]	Gallery game: Data collection on cognitive abilities	Smartphone app	Yes	Yes	Early (pilot)	Efficacy
**Decision support**						
Bogza et al (2020); Canada [71]	Web-based decision aid for MCI intervention: Aid people in becoming better informed and involved in decision-making	Website	No	No	Early (prototype)	Usability
Ekstract et al (2017); United States [72]	APOE^b^ genetic testing decision aid: Educate people about APOE testing and help them decide whether to undergo it	Website	Yes	Yes	Mid (demonstration)	Effectiveness

a MCI: mild cognitive impairment.

b APOE: apolipoprotein E.

### Purpose of Digital Tools

We identified 4 main categories of tools (Table 2). The first category was tools aimed at dementia prevention, including primary prevention (14/39, 36%) and secondary prevention (11/39, 28%). Primary prevention–related tools generally comprised lifestyle-related risk reduction, whereas secondary prevention–related tools generally focused on enhancing cognitive functioning. Both primary and secondary prevention tools did so via single-domain interventions, such as cognitive training [36,37,43,45,46,48-51,53,57,59] (primary prevention: 8/15, 53%; secondary prevention: 4/10, 40%), or multidomain interventions, such as improving physical activity, social activity, mental activity, lifestyle, and attitude [34,35,38,39,41,44,47,52,54-56,58,73] (primary prevention: 7/15, 47%; secondary prevention: 6/10, 60%). Second, daily life support tools (8/39, 21%) frequently aimed to help people manage symptoms and/or decrease the impact of cognitive complaints on daily life, for instance, via memory support or enhancing quality of life. The main aim of the self-administered screening-related tools (4/39, 10%) was to assess current cognitive functioning and/or the risk of developing AD/dementia. Finally, decision aids (2/39, 5%) had an overarching goal of supporting shared decision-making regarding certain health care decisions (eg, via preference elicitation).

### Developmental Stage of Tools

Most tools were in an early maturity stage of evaluation/testing (31/39, 80%) [32], comprising preprototyping (1/35, 3%), prototyping (15/35, 43%), or pilot testing (19/35, 54%), and not yet in the stage of demonstration in uncontrolled conditions (Table 2). Overall, the stage of evaluation [32] mainly comprised efficacy testing (18/40, 45%), followed by usability testing (12/40, 30%) and feasibility testing (10/40, 25%; Table 2). Usability and feasibility testing were often combined with each other, whereas efficacy or effectiveness testing were usually performed independently. Some studies combined multiple forms of maturity and evaluation testing (eg, prototyping and pilot testing or feasibility and usability testing), explaining why there are more total testing methods than papers. Eight tools were in a mid-maturity stage and evaluation stage (8/39, 21%).

### Future Directions

Five main themes related to future research directions were identified (Table 3). First, many suggested directions were related to early tool development, whereby authors suggested an array of directions surrounding future early development steps or recommended early development steps in general, such as the need for (further) prototyping or developing a digital tool via co-design (Table 3).

**Table 3.** Themes of future (research) directions.

Main category and subcategories		Mentioned by
**Development in early stage of maturity**		
	Need for (further) prototyping (ie, design adjustments)	[34,35,43-45,47,57,60,62,63,65,69]
	Add personalization (ie, tailored approaches to enhance engagement or personalized presentation of intervention)	[35,53,60,62,63]
	Applying technological improvements (ie, improving reliability and ensuring product updates)	[36,55,72]
	Investigate gamification of intervention programs	[37]
	Develop via co-creation and co-design with end users	[40,63]
**Development in early stage of evaluation**		
	Need for feasibility research (ie, contextual research)	[42,68]
	Improve usability via user testing (ie, validation, user experience testing, assess over time)	[41,55,59,63,71]
	Adjust protocol/refinement of training material	[51,61]
	Need for efficacy testing	[40,46,47,50,52,54,58,59]
**Development in the mid stage of maturity**		
	Perform implementation research (ie, long-term adoption or test in health care settings)	[37,44,53,56,60,64,71]
**Development in the mid stage of evaluation**		
	Need for effectiveness testing	[45,46,48,56,60,65-67,71]
**Development in advanced stage of maturity**		
	Test in multiple settings (ie, comparing intervention settings)	[50]
**Diversity, equity, and inclusivity**		
	Increase cross-cultural relevance (ie, validating cross-cultural relevance)	[35,38,41,55,63,70]
	Ensure diverse study population (ie, representative study samples to assess accessibility and acceptability)	[36,39,41,42,44,50,55,56,58,63,65,72]
**Study advancements**		
	Standardize participant recruitment criteria	[41]
	Conduct large-scale studies	[34,38-40,51,56-58,67]
	Apply methodological improvements (eg, small sample for inferential statistics; broad definition of mild cognitive impairment)	[50,54]
	Use different analyses (eg, explore age-related differences; assess effect on global motor activities and possible negative mental effects due to uncontrolled cognitive training activities)	[49,50,57,58]
	Outcome measure assessment (ie, develop methods to assess success)	[37]
	Retention (ie, improve participant retention)	[36,40,41]
	Conduct longitudinal studies	[37,41,46,51,55,62,66,72]
	Address study limitations (eg, evaluate and refine intervention strategies; prioritize at-risk populations; address bias)	[38,40,58]
	Conduct randomized controlled trial	[38,39,52,56,57,59,62]

Second, some studies comprised a digital tool in the early development stage and looked forward to preparing for the mid-maturity stage. These studies, for instance, contained future directions surrounding implementation research or mentioned the need for efficacy testing of their digital tool (Table 3). Third, some studies even mentioned future directions regarding advanced development stages, such as digital tool testing in multiple settings. Fourth, themes regarding the diversity and inclusivity of both the study population and the content of the tool were mentioned as future directions (Table 3). Finally, multiple subthemes regarding study advancements were mentioned. These were usually mentioned after reflecting on the study and its limitations, such as the need for methodological changes. Some of these study advancement directions were mentioned in light of next steps needed, such as longitudinal randomized controlled trial testing (Table 3).

## Discussion

### Principal Findings

This scoping review sought to create a state-of-the-art overview of digital tools for people without an Alzheimer’s disease (AD) or dementia diagnosis as currently reported in the scientific literature. Our scoping review shows a considerable number of tools that people without an AD or dementia diagnosis can use by themselves, with almost 70% of articles published in the last 5 years (27/39, 69.2%). Digital tools focused on training cognitive functioning, improving lifestyle, improving daily life functioning, assessing current cognitive functioning and/or the risk of developing AD or dementia, and decision support regarding brain-related health care. Yet, the majority of digital tools reported on were directed at primary or secondary prevention, including both single-domain and multidomain interventions. These findings underscore the recent trend toward dementia prevention efforts in dementia-related health care [11-13,74].

We found no digital tools that calculate an individual’s dementia risk estimate and few self-administered screening-related tools that people can use by themselves. The shift toward early prediction and diagnosis may be too recent to already have resulted in scientific reports on these types of digital tools [8]. In addition, this shift toward earlier disease phases and greater responsibility for individuals to take an active role in their own brain health and dementia prevention comes with new ethical, legal, and societal implications such as the risk of misinformation, potentially enlarging existing health inequities, the balance between responsibility and autonomy, data privacy issues, ownership of sensitive health data, conflict with other non–health-related values people hold in life, the risk of increasing anxiety, stigmatization, medicalization, and increased social pressure on the individual [75-78]. These issues may need to be addressed first in order to develop prediction and self-administered screening tools that are safe and acceptable to use by people themselves, without professional involvement or assistance. Nevertheless, some digital prediction and diagnostic tools are already available for memory clinic physicians [79-82]. Given the trend toward greater individual responsibility and timely diagnosis, we expect that more tools will emerge in the coming years that cater toward individuals with cognitive complaints or those at risk as the primary end users.

We identified fewer digital tools to support daily life for people with SCD or MCI than expected [83,84]. For instance, only 2 decision-support tools could be identified in academic literature in this review. In other populations, research shows that decision-support tools may enhance shared decision-making processes, increase patient knowledge and empowerment, reduce decisional conflict, and improve patient-provider communication [85,86]. Therefore, developing decision-support tools may also become increasingly relevant for the field of AD and dementia as recent biomedical developments allow for early diagnosis, dementia risk prediction, and dementia prevention, as this comes with increasingly complex decisions to be made by individuals and their care partners (for instance, what treatment burden and risk is someone willing to accept) [87]. We did not find any wayfinding digital tools that people with MCI could use as end users. We found some articles on wayfinding systems; however, we had to exclude these as the person with MCI was not the main end user. These finding thus highlight a potential research gap and opportunity for future support.

Most digital tools for cognitively unimpaired people or people with SCD or MCI in the context of AD or dementia as described in the literature are still in (early) development, in which usefulness, feasibility, and initial efficacy are investigated, rather than in a phase of widespread implementation and effectiveness testing. Primary dementia prevention tools seem to be slightly further in terms of maturity and evaluation, indicated by 5 articles that report on tool effectiveness [37,39,43,46,49]. Our overview of digital tools, including their stages of maturity and evaluation, shows that the evidence gathered for digital tools for cognitively unimpaired people or people with SCD or MCI in the context of AD or dementia in the literature is limited. Health care policies should therefore not yet be based on the availability of evidence-based digital tools.

With regard to study samples, our findings show an overrepresentation of women aged 60 years and older and underrepresentation of diverse study populations regarding digital literacy and ethnicity. The World Health Organization states that digital technology plays a crucial role in achieving universal health coverage and adds that digital tools are essential for promoting health and serving the disadvantaged [88,89]. Development and evaluation processes of digital tools should thus take an inclusive approach, with representative study samples when investigating the usability and feasibility of digital tools [90]. Study samples in research on digital tools in the context of dementia prevention and self-administered screening should be diverse in terms of sex, gender, race and/or ethnicity, religion, disability, educational level, socioeconomic position, and people with marginalized status (eg, Indigenous Peoples), as these characteristics can affect a person’s risk of dementia [74,91]. In addition, it is important to include people with varying levels of digital health literacy (DHL). DHL encompasses the ability of individuals to seek, comprehend, and apply health information from digital sources, using various technologies and digital platforms [92]. It involves the skills and knowledge required to navigate, evaluate, and use digital tools, websites, and apps to access health-related information and services [92]. Although commonly used methods to assess DHL skills are available [93,94], we found that current research on digital tools for dementia prevention and self-administered screening lacks the reporting of such DHL skills. Approximately one-quarter of papers discussed the computer-, Wii-, and smartphone-related expertise of participants; however, these participants were often digitally proficient. Additionally, around 70% of papers did not report anything on the DHL skills of their participants. Moreover, people can experience barriers, such as difficulty navigating the system, that hamper the successful use and adoption of digital tools due to a chronic disease or general aging processes [95-97]. These barriers are well-known, but people with low DHL nevertheless remain underrepresented in research. In our ongoing research, we emphasize the importance of including people with varying educational attainment, digital proficiency, and digital acceptability as these factors seem to influence people’s intention to use and satisfaction with digital tools for dementia prevention (see [98]).

The results from this scoping review also highlight the importance of oversampling culturally diverse and underrepresented populations during recruitment to result in more diverse study samples. Other recommendations regarding inclusivity include engaging in dedicated usability testing sessions and using inclusive design guidelines (eg, Web Content Accessibility Guidelines and the upcoming European Accessibility Act requirements) [99-102], as well as engaging in co-design, in which a representative group of (potential) end users directly cooperate in a creative way with developers and researchers throughout the entire (tool) design process [103]. Specific co-design methods exist for working together with people with cognitive complaints and/or dementia, such as carefully introducing new topics, using probes to involve people in abstract thinking, guiding caregivers toward a supporting role, and personalizing the length of an interview or session [104]. Tools are developed that address a need of end users and are acceptable, usable, and inclusive for end users. Finally, from an accessibility perspective, it is recommended to offer digital tools as part of a larger intervention containing (nondigital) alternatives.

### Strengths and Limitations

Among the strengths of this scoping review is the thorough process of article inclusion, conducted by 3 reviewers, ensuring researcher triangulation. As for limitations, we only included Dutch and English articles. We also may have missed some articles, since we did not explicitly search for digital tools on cognitive training or on specific dementia-related risk factors, such as hearing loss prevention or high blood pressure. In our search query, we also searched for “diagnostic tools” and “screening” instead of “self-administered screening tools,” which may have resulted in the exclusion of relevant articles since they were not identified through the search query. Moreover, we did not perform snowballing. We included tools on dementia prevention, daily life support, AD/dementia diagnosis and risk assessment, and decision support. We may have missed some tools that improve brain health but are not explicitly linked to brain health in the context of AD or dementia. For instance, digital tools targeting lifestyle in general, word games, or exergames may also help to reduce dementia risk, but these did not appear in our search results if they were not explicitly linked to AD or dementia. Likewise, certain assistive technologies and tools targeting people with dementia and their informal caregivers might also be useful in earlier disease stages, such as lifestyle-monitoring systems [105]. We did not include these articles since we focused on cognitively unimpaired people or people with SCD or MCI in the context of AD or dementia.

### Future Work

A variety of future (research) directions were reported in the included papers, mainly focusing on improvements in the early development stages, such as the need for (further) efficacy or feasibility testing and the need for (further) prototyping. Many included papers also mentioned that they would focus on effectiveness testing in future research in a real-world setting and/or in large-scale clinical randomized controlled trials. In addition, implementation research is considered crucial to translate digital tools into practice [106,107]. Previous research shows that successful implementation of digital tools in the AD context proves to be difficult [19,22,23]. Some factors hindering implementation might relate to common characteristics of the target group of people with or at high risk for AD/dementia, such as older age, cognitive impairment, impaired hearing, problems with vision, and comorbidities [3,25,26]. Personal factors of the person at risk, such as attitudes and beliefs, might also influence people’s intention to use digital tools for health (care) purposes [108-110]. Moreover, systematic implementation issues on an interpersonal level (eg, social influences of others on digital tool use) and systemic level (eg, facilitating health care policies) should be addressed [111]. If these factors are not taken into account, digital tools may only benefit those who are already better off and structural inequities may reinforce themselves, enlarging health disparities [112]. Thus, an overarching examination of factors associated with implementation success of digital tools in the context of cognitively unimpaired people or people with SCD or MCI in the context of AD or dementia is needed to allow for successful implementation in practice for all. This scoping review captured tools described in the academic literature. It must be noted that some digital tools included in our study as early stage of maturity and evaluation are currently already in mid-mature phases among other populations. However, it may be that their follow-up development has not been reported in academic literature or that follow-up papers assessing the mid-maturity stage of maturity and evaluation did not show up in our search results (eg, CogniFit [113]). It is also likely that there is a broad variety of digital tools on the market that are not reported on in academic literature. This implies that even though we found that the majority of digital tools as reported on in academic literature are considered to be in early maturity stages, the actual stage of maturity and evaluation may be different than reported on in academic literature. Future research may combine these findings from academic literature with digital tools as reported on in gray literature for a more comprehensive overview, focusing on performance accuracy, stage of evaluation, and stage of maturity. Future research could create an overview of digital tools developed in the context of AD or dementia for cognitively unimpaired people or people with SCD or MCI reported on outside of academic literature.

### Conclusion

This scoping review shows that the majority of digital tools for cognitively unimpaired people or people with SCD or MCI reported on in academic AD and dementia literature comprise primary and secondary dementia prevention–related tools (64%). Tools that support people who do not have AD or dementia in activities of daily living are also common (20%). Digital tools are tested among people who vary in cognitive abilities, but reported studies were skewed toward highly educated women aged 60 years and older. Few papers reported on the ethnicity and digital literacy of their study population. Almost 80% of digital tools as reported on in academic literature are in (early) development, comprising early stages of maturity (eg, preprototyping, prototyping, or pilot testing) and early stages of evaluation (eg, efficacy testing, usability testing, and feasibility testing). The scientific evidence gathered for digital tools for people without an AD or dementia diagnosis is limited. Future (research) directions comprise the need for ensuring diversity, equity, and inclusion, as well as the need for implementation research. We therefore encourage further research and policy development to support the transition of promising tools from the early development stage to later stages, thereby allowing for increased use among people without an AD or dementia diagnosis.

## Supplementary material

10.2196/64862 Multimedia Appendix 1 Supplementary materials.

10.2196/64862 Checklist 1 PRISMA checklist.

## Abbreviations

AD: Alzheimer disease
DHL: digital health literacy
MCI: mild cognitive impairment
PRISMA: Preferred Reporting Items for Systematic Reviews and Meta-Analyses
SCD: subjective cognitive decline
