# Supplementary material for: Digital Tools for People Without an Alzheimer Disease or Dementia Diagnosis: Scoping Review
Source: J Med Internet Res. 2025 Aug 25;27:e64862. doi: 10.2196/64862 (PMC12377699; doi:10.2196/64862)
Supplement: Multimedia Appendix 1 [file jmir-v27-e64862-s001.docx]

**Multimedia Appendix 1**

**Final search queries**

1. **Pubmed**

((Neurocognitive Disorders [MeSH Terms]) OR (Alzheimer Disease [MeSH Terms]) OR (Cognitive Dysfunction[MeSH Terms]) OR (Cognition Disorders [MeSH Terms]) OR (Memory Disorders[MeSH Terms]) OR ADRD OR Alzheimer*) AND ((Smartphone [MeSH Terms]) OR (Cell Phone [MeSH Terms]) OR (Cell Phone Use [MeSH Terms]) OR (mobile applications [MeSH Terms]) OR (telemedicine [MeSH Terms]) OR (telephone [MeSH Terms]) OR handheld com- puter* OR cell phone* OR mHealth* OR m-Health* OR eHealth* OR e-Health* OR telemedicine OR tele-medicine OR telehealth OR tele-health OR tele* mobile device* OR mobile phone* OR mobile app* OR mobile application* OR educational video* OR personal digital assistant OR smartphone* OR mobile telephone* Decision making, shared OR digital OR digitized OR web tool OR smartphone application*) AND ((Prognosis [MeSH Terms]) OR (Symptom Assessment [MeSH Terms]) OR (Risk Assessment [MeSH Terms]) OR (Risk Management [MeSH Terms]) OR (Diagnostic Self Evaluation [MeSH Terms]) OR (Diagnostic Screening Programs [MeSH Terms]) OR (Diagnostic Services [MeSH Terms]) OR (Physician-Patient Relations[MeSH]) OR (Patient participation [MeSH]) OR (Decision making, shared [MeSH]) OR (Patient education as topic [MeSH]) OR assistive technology* OR Doctor-patient communication* OR patient education* OR awareness* OR management* OR messaging* OR ambient assisted living* OR technology solution* OR software OR wearable sensors* OR smart home* OR telemonitoring* OR global positioning system)

1. **IEEE Xplore**

((”All Metadata”:Neurocognitive Disorders) OR (”All Metadata”:Alzheimer Disease ) OR (”All Metadata”:Cognitive Dysfunction) OR (”All Metadata”:Cognition Disorders) OR (”All Metadata”:Memory Disorders) OR (”All Meta- data”:Alzheimer*)) AND ((”All Metadata”:Smartphone ) OR (”All Metadata”:Cell Phone) OR (”All Metadata”:Cell Phone Use) OR (”All Metadata”:mobile applications) OR (”All Metadata”:telemedicine) OR (”All Metadata”:telephone

) OR (”All Metadata”:cell phone) OR (”All Metadata”:mHealth* ) OR (”All Metadata”:eHealth*) OR (”All Metadata”:mobile device) OR (”All Metadata”:mobile phone) OR (”All Metadata”:mobile app) OR (”All Meta- data”:mobile application*) OR (”All Metadata”:telemedicine) OR (”All Metadata”:mobile device) OR (”All Meta- data”:educational video) OR (”All Metadata”:personal digital assistant) OR (”All Metadata”:online tool) OR (”All Metadata”:web tool) OR (”All Metadata”:handheld computer) OR (”All Metadata”:digital) OR (”All Meta- data”:digitized) OR (”All Metadata”:smartphone application*)) AND ((”All Metadata”:Prognosis ) OR (”All Metadata”:Symptom Assessment) OR (”All Metadata”:Risk Assessment) OR (”All Metadata”:Risk Management) OR (”All Metadata”:Diagnostic Self Evaluation) OR (”All Metadata”: Diagnostic Screening Programs) OR (”All Metadata”:Physician-Patient Relations) OR (”All Metadata”:Patient Participation) OR (”All Metadata”:Patient education as topic) OR (”All Metadata”:Doctor-patient communication*) OR (”All Metadata”:management) OR (”All Metadata”:message*) OR (”All Metadata”: assistive technology) OR (”All Metadata”:predict*) OR (”All

Metadata”:screening) OR (”All Metadata”:communication) OR (”All Metadata”:diagnosis) OR (”All Metadata”:assessment) OR (”All Metadata”:ambient assisted living) OR (”All Metadata”: technology solution) OR (”All Metadata”:software)

OR (”All Metadata”:wearable sensors) OR (”All Metadata”:smart home) OR (”All Metadata”:telemonitoring) OR (”All Metadata”:global positioning system))

1. **Web of Science**

(TS=(Alzheimer Disease*) OR TS=(ADRD) OR TS=(Alzheimer*) OR TS=(Dementia*) OR TS =(cognitive dys- function) OR TS = (neurocognitive disorder)) AND ((TS=(Smartphone*) OR TS=(Cell Phone*) OR TS=(handheld computer*) OR TS=(mHealth*)) OR TS=( eHealth*) OR TS=(telemedicine*) OR TS=(mobile phone*) OR TS=(mobile app*) OR TS=(web tool*) OR TS=(smartphone application*) OR TS=(digital *) OR TS=(telehealth*) OR TS=(tele*) OR TS = (wearable sensors*) OR TS=(online tool*)) AND (TS = (Doctor-patient communication*) OR TS = (assistive technology*) OR TS=(risk management*) OR TS = (Patient Education*)OR TS=(screening*) OR TS=(prevention*) OR TS = (Symptom Assessment*) OR TS = (risk assessment*)OR TS=((Health) NEAR/1 (info* OR education*)) OR TS=(Diagnostic Screening Programs*) OR TS=(Decision making, shared*) OR TS=( information dissemination*) OR TS = (physician-patient communication) OR TS = (physician-patient relation*) OR TS = (patient participation) OR TS =(predicti*) OR TS = (prognosis)

1. **OVID**

| **Number** | **Query** | **Results from 21 Jan 2023** |
| --- | --- | --- |
| 1 | Alzheimer Disease/ | 114,776 |
| 2 | (alzheimer* or alzeimer*).tw. | 215,586 |
| 3 | (dementia adj2 (senile or presenile)).tw. | 4,416 |
| 4 | (cortical adj4 sclerosis).tw. | 1,477 |
| 5 | 1 or 2 or 3 or 4 | 231,894 |
| 6 | (telehealth or telemedicine or teleconsultat* or telerehab* or ehealth or e health  or ((digital* or tele* or virtual*) adj3 (explain* or care or communicat* or  inform* or educat* or counsel* or consult*))).ti,ab,kf. | 65,068 |
| 7 | exp internet/ or exp computers handheld/ or mobile applications/ or (handheld  computer* or cell phone* or mHealth* or m-Health* or eHealth* or e-Health* or telemedicine or tele-medicine or telehealth or tele-health or tele* mobile device* or mobile phone* or mobile app* or mobile application* or educational video*or personal digital assistant or smartphone* or mobile telephone* or online tool*  or digital or digitized or web tool or smartphone application*).ti,ab,kf. | 347,534 |
| 8 | (app or apps or blog* or elearn* or e-learn* or facetime or face time or forum* or  helpline* or hotline* or internet* or ipad* or iphone*or online or mobile phone* or personal digital assistant* or pocket pc* or skype* or smartphone* or smart phone* or social media or social network* or sms or text messag* or video* or  web*).ti. | 154,719 |
| 9 | 6 or 7 or 8 | 457,801 |
| 10 | (Delivery of health information or Health information or Health education).tw. | 98,533 |
| 11 | ((inform* or knowledge) adj3 (explain* or search* or seek* or access* or provi-  sion* or provid* or acquisition or obtain* or acquir*)).ti,ab,kf. | 427,025 |
| 12 | exp Patient Education as Topic/ or exp Patient Education Handout/ or (exp  communication/ and exp Telemedicine/) or Information Seeking Behavior/ or  exp Consumer Health Information/ or exp health education/ | 270,845 |
| 13 | exp Risk Assessment/ or Risk Management/ or Risk Reduction Behavior/ | 319,881 |
| 14 | information centers/ or information services/ or information dissemination/ | 34,937 |
| 15 | (assistive technology* or Doctor-patient communication* or patient education*  or awareness* or management* or messaging*).ti,ab,kf. | 1,730,169 |
| 16 | 10 or 11 or 12 or 13 or 14 or 15 | 2,671,365 |
| 17 | prevent*.tw. | 2,398,893 |
| 18 | diagnos*.tw. | 3,850,289 |
| 19 | exp Diagnosis/ | 9,255,852 |
| 20 | 18 or 19 | 11,664,635 |
| 21 | 16 or 17 or 20 | 14,600,919 |
| 22 | animals/ not humans/ | 5,050,981 |
| 23 | 5 and 9 and 21 | 1,768 |
| 24 | 23 not 22 | 1,416 |
| 25 | limit 24 to english language | 1,383 |
| 26 | limit 25 to yr=”2013 -Current” | 877 |

**Data charting form**

| **Categories** | **Description** |
| --- | --- |
| Author | The first author of the study. |
| Year | The publication year of the study. |
| Location | The country where the study was performed. |
| Aim of tool | The aim of the tool. |
| Type of tool | Brief description of the form of the tool (e.g., a website or tablet application). |
| Self-guidance | Whether or not healthcare providers or researchers assisted tool use during the study or whether tool use was mainly self-guided. |
| Place of use | The setting in which the tool was used in the study (e.g., in the home-setting or in a clinical setting). |
| Study aim | The aim of the study. |
| Conclusion | The conclusion of the study. |
| Participants | The number of participants. |
| Cognitive status | The cognitive status of study participants. |
| Age | The age of study participants. |
| Sex | The sex of study participants. |
| Ethnicity | The ethnicity of study participants. |
| Educational level | The educational level of study participants. |
| Digital literacy | The digital literacy of study participants. |
| Stage of maturity | The stage of developmental maturity of the tool.[14] |
| Stage of evaluation | The stage of impact evaluation of the tool.[14] |
| Future directions | Recommendations for future research that were mentioned by the authors. |
